# Editorial: Expert opinions in protein bioinformatics: 2022

**DOI:** 10.3389/fbinf.2023.1338560

**Published:** 2024-01-05

**Authors:** Daisuke Kihara

**Affiliations:** Department of Biological Sciences, Department of Computer Science, Purdue University, West Lafayette, IN, United States

**Keywords:** protein bioinformatics, editorial, protein structure prediction, general purpose modeling methods, AlphaFold2

In 2021, protein bioinformatics underwent a profound and irreversible transformation with the publication of AlphaFold2, a method for predicting protein tertiary structures. AlphaFold2 (Jumper et al., 2021) demonstrated significantly higher accuracy compared to existing methods during the 14th Critical Assessment of Techniques for Protein Structure Prediction (CASP14). Notably, its accuracy often rivals that of experimental structure determination techniques like X-ray crystallography, which has proved to be exceptionally valuable in various practical applications.

An additional noteworthy aspect of AlphaFold2 is that it represents the first successful end-to-end deep neural network for protein structure prediction. This achievement is particularly remarkable given that protein structure prediction has long been recognized as one of the most challenging problems in bioinformatics and computational biophysics. Unlike existing methods with complex architectures involving intricate steps in a computational modeling pipeline, AlphaFold2 demonstrated that the entire process can be encapsulated within a single neural network.

The success of Alphafold2 is resulted from convergence of crucial elements, including the maturation of the protein structure prediction problem, the availability of a substantial dataset, the rapid advancements in deep learning, and the presence of adequate computational resources. This also indicates that the same approach would be possible for other molecular modeling problems, too.

Following AlphaFold2, a cascade of related developments ensued, spanning protein docking prediction, algorithms for protein design and drug design, RNA structure prediction, and predictions of the effects of missense mutations, all using deep learning. Consequently, the emergence of AlphaFold2 will be remembered not only as a singular achievement but also as the beginning of the end for the development of general-purpose modeling methods that aim to be applied to any proteins, drugs, RNA, and complexes.

In the era of the end of general-purpose modeling problems, an increasing emphasis will be placed on the accurate solutions to individual, fine-grained, and specific tasks. This compilation serves as a testament to the significant research topics that define the post-general-purpose problems era. Gomez et al. explored the applicability of AlphaFold2 models within the context of force spectroscopy experiments (Gomez et al.). Chang et al. delved into the key factors crucial for the successful computational design of peptides, highlighting an important application of protein design methods (Chang et al.). The article by Neumann et al. the Ben-Hur group specifically addresses the importance of the appropriate selection of negative examples in predicting host-pathogen protein interactions (Neumann et al.). Lastly, Jeffery provides insights into the various challenges that make protein function prediction a complex task (Jeffery).

As we navigate the landscape of the post-general-purpose method development, the contributions highlighted in this Research Topic reflect the profound impact and continued evolution of methodologies addressing specific molecular tasks. The success of future protein bioinformatics projects will hinge on the seamless integration of a detailed understanding of biological problems and background, coupled with the proper application of machine learning methods, to accomplish specific tasks.
